# Expression and Functionality of Connexin-Based Channels in Human Liver Cancer Cell Lines

**DOI:** 10.3390/ijms222212187

**Published:** 2021-11-10

**Authors:** Kaat Leroy, Cícero Júlio Silva Costa, Alanah Pieters, Bruna dos Santos Rodrigues, Raf Van Campenhout, Axelle Cooreman, Andrés Tabernilla, Bruno Cogliati, Mathieu Vinken

**Affiliations:** 1 Entity of In Vitro Toxicology and Dermato-Cosmetology, Department of Pharmaceutical and Pharmacological Sciences, Vrije Universiteit Brussel, Laarbeeklaan 103, 1090 Brussels, Belgium; kaat.leroy@vub.be (K.L.); alanah.pieters@vub.be (A.P.); bruna.dos.santos.rodrigues@vub.be (B.d.S.R.); raf.van.campenhout@vub.be (R.V.C.); axelle.cooreman@vub.be (A.C.); andres.tabernilla.garcia@vub.be (A.T.); 2 Department of Pathology, School of Veterinary Medicine and Animal Science, University of São Paulo, Av. Prof. Dr. Orlando Marques de Paiva 87, Cidade Universitária, São Paulo 05508-270, Brazil; cicerocosta@usp.br (C.J.S.C.); bcogliati@usp.br (B.C.)

**Keywords:** connexin, gap junction, liver cancer, cell line, in vitro

## Abstract

Liver cancer cell lines are frequently used in vitro tools to test candidate anti-cancer agents as well as to elucidate mechanisms of liver carcinogenesis. Among such mechanisms is cellular communication mediated by connexin-based gap junctions. The present study investigated changes in connexin expression and gap junction functionality in liver cancer in vitro. For this purpose, seven human liver cancer cell lines, as well as primary human hepatocytes, were subjected to connexin and gap junction analysis at the transcriptional, translational and activity level. Real-time quantitative reverse transcription polymerase chain reaction analysis showed enhanced expression of connexin43 in the majority of liver cancer cell lines at the expense of connexin32 and connexin26. Some of these changes were paralleled at the protein level, as evidenced by immunoblot analysis and in situ immunocytochemistry. Gap junctional intercellular communication, assessed by the scrape loading/dye transfer assay, was generally low in all liver cancer cell lines. Collectively, these results provide a full scenario of modifications in hepatocyte connexin production and gap junction activity in cultured liver cancer cell lines. The findings may be valuable for the selection of neoplastic hepatocytes for future mechanistic investigation and testing of anti-cancer drugs that target connexins and their channels.

## 1. Introduction

Liver cancer, mainly represented by hepatocellular carcinoma (HCC), is the second and sixth most common cause of cancer death among men and women, respectively [1]. Primary liver cancer is associated with a wide variety of causes, including chemical, viral and dietary factors [2,3]. Many laboratories worldwide have devoted their work to the study of the mechanisms underlying liver cancer, as well as to the characterization of potential targets for the development of new liver cancer therapeutics [4]. A multitude of experimental systems and models has been used throughout this research [5,6,7]. While some animal models of liver cancer can provide a good reflection of the corresponding human disease and thus have high translational value, they come with a number of issues [7,8,9,10]. In this respect, besides the obvious ethical constraints, animal models are not always well-fit for mechanistic investigation because of their complex dynamic nature [11]. In vitro models seem better suited for this purpose, as they provide a more controllable experimental setting that allows the dissection of specific signaling pathways [5,6,8].

Direct intercellular signaling is mainly mediated by gap junctions [12]. Gap junctions establish a pathway for the exchange of small and hydrophilic molecules and ions between neighboring cells, a flux denoted gap junctional intercellular communication (GJIC). Gap junctions consist of two hemichannels of adjacent cells, which in turn are built by six connexin (Cx) proteins [12]. Connexins are expressed in a cell-type-specific fashion [13]. In healthy liver, the most abundantly expressed connexin species by hepatocytes is Cx32 next to small quantities of Cx26 [14]. By contrast, non-parenchymal cell types in the liver, including Kupffer cells [15] and stellate cells [16], mainly produce Cx43 [17]. During chronic liver disease, however, which may burgeon into the onset of liver cancer, a switch takes place in the connexin expression pattern [17]. Specifically, Cx43 becomes increasingly detectable in the HCC tissue of human patients suffering from liver cancer and experimental animal models not only in non-parenchymal liver cells, but equally in neoplastic hepatocytes [18,19,20,21,22]. This is accompanied by a reduction in the expression of Cx26 [21,22,23] and in particular that of Cx32 [21,23,24], as well as an overall deterioration of GJIC [17].

Liver cancer cell lines are frequently used in vitro tools to test candidate anti-cancer agents as well as to elucidate mechanisms of liver carcinogenesis [25], such as cellular communication mediated by connexin-based gap junctions [13]. The present study is set up to investigate connexin expression and gap junction functionality in liver cancer in vitro compared to healthy primary human hepatocytes (PHH). To this end, seven human cancer liver cell lines, including six from HCC and one of adenocarcinoma origin, are subjected to analysis of connexin mRNA and protein abundance, as well as of subcellular localization. This is aligned by measurement of the activity of gap junctions. This will provide a full scenario of modifications in connexin expression and gap junction activity in cultured liver cancer cell lines, which may be valuable for the selection of neoplastic hepatocytes for future mechanistic investigation and testing of anti-cancer drugs targeting connexin proteins.

## 2. Results

### 2.1. Selection of Liver Cancer Cell Lines

In order to assess connexin expression in liver cancer in vitro, a panel of seven cancer cell lines was selected (Table 1). The panel contained six HCC cell lines (C3A, SNU-449, SNU-423, SNU-387, SNU-475 and PLC/PRF/5) and one adenocarcinoma cell line (SK-HEP-1), primarily derived from Caucasian or Asian male patients.

**Figure 1:**
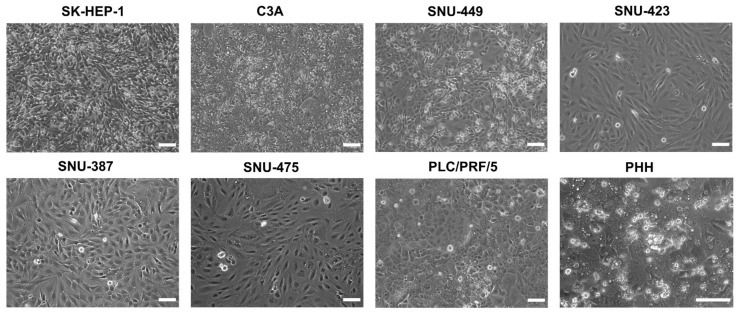
Phase contrast images of liver cancer cell lines and primary human hepatocytes (PHH) at 100% confluence. All assays and extractions were performed when liver cancer cell lines reached 100% confluence. Phase contrast images were taken on a Nikon Eclipse T*i*-S microscope (Nikon, Tokyo, Japan) with a 10× (liver cancer cell lines) or 20× objective (PHH). Scale bar = 100 µm.

The cell lines all varied in differentiation status and metastatic potential. In this respect, SK-HEP-1 cells are highly metastatic and display mesenchymal stem cell characteristics [26], while C3A cells, which are derived from the commonly used HepG2 cell line, are more differentiated and have retained some metabolic activity [27]. In contrast to C3A cells, the SNU cell lines are less differentiated, more invasive and mesenchymal-like [28]. In order to fully characterize the expression of connexins in HCC, the expression levels were compared to PHH, which represented the normal homeostatic state. Hepatocytes are typically polygonal and can have 1–3 nuclei [29,30]. During carcinogenesis, their morphology changes to a more fibroblast-like phenotype [30]. This was clearly seen in this study (Figure 1).

Morphology pictures were taken during the exponential growth phase (Figure S1) of the cancer cell lines or at 100% confluence (Figure 1) and at the last day of the PHH sandwich cultivation period (Figure 1 and Figure S1). Most cancer cell lines displayed a spindle form and appeared to be larger and/or flatter compared to PHH (Figure 1 and Figure S1). The C3A cell line, which is known to be more differentiated [27], exhibited a morphology that is distinct from the other cancer cell lines. The C3A cells were less elongated and had partly retained the polygonal shape and thickness of PHH, which was also the case for PLC/PRF/5 (Figure S1). The retained polygonal shape of PLC/PRF/5 cells was clearer at 100% confluence compared to their appearance during the exponential growth phase ((Figure 1 and Figure S1). In addition, C3A and PLC/PRF/5 cells tended to grow in clusters compared to the other more fibroblast-like cell lines that grew in a looser pattern (Figure S1).

### 2.2. Connexin Gene Expression in Liver Cancer Cell Lines

**Figure 2:**
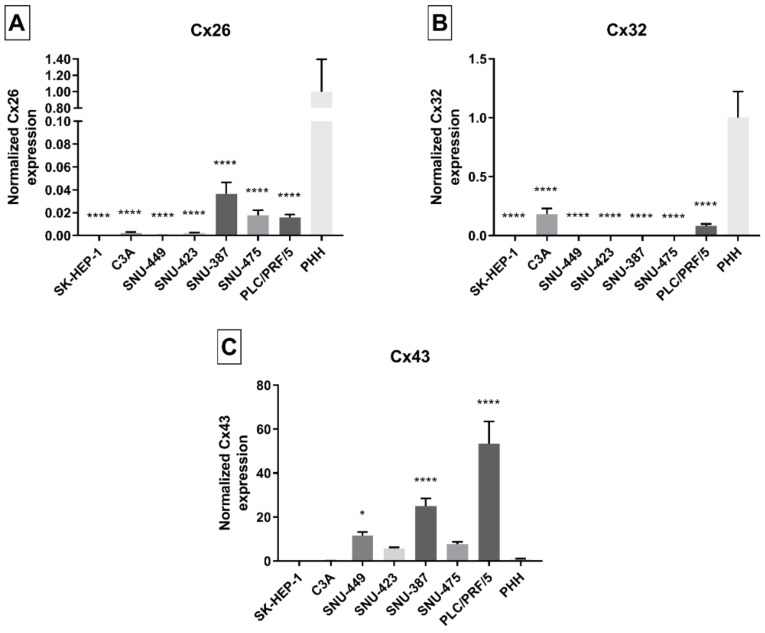
*Cx26* (**A**), *Cx32* (**B**) and *Cx43* (**C**) gene expression in liver cancer cell lines and primary human hepatocytes (PHH). Cancer cell lines were grown to 100% confluence, while PHH were used in suspension when total RNA was extracted (*n* = 1, *N* = 3). Subsequently, real-time quantitative reverse transcription polymerase chain reaction (RT-qPCR) analysis was performed. Relative alterations compared to PHH were calculated according to the Pfaffl method in qbase^+^ (Biogazelle, Gent, Belgium). Data are expressed as mean ± standard deviation with * *p* ≤ 0.05 and **** *p* ≤ 0.0001 compared to the PHH control.

In vivo, human hepatocytes mainly produce Cx32, and to a lesser extent Cx26, which account for 90% and 5% of connexin protein expression, respectively [31]. While Cx32 is ubiquitously expressed [32], *Cx26* mRNA is more restricted to the periportal areas [33]. Cx43, on the other hand, is expressed by non-parenchymal cells, such as stellate cells and Kupffer cells [16,34,35,36]. During liver disease, in particular upon acute inflammation, *Cx32* mRNA expression is decreased because of increased degradation [37]. In HCC, *Cx26* [38] and *Cx32* [19,38] gene expression is downregulated, while *Cx43* mRNA production becomes promoted [19,20]. However, other studies have shown opposite changes for *Cx43* mRNA expression [38] or no changes for *Cx32* gene expression [20,21]. Real-time quantitative reverse transcription polymerase chain reaction (RT-qPCR) analysis in this study detected all connexin species in PHH. Data collected from 100% confluent cancer cell line cultures and PHH confirmed that *Cx26* (Figure 2A) and *Cx32* (Figure 2B) mRNA quantities were strongly decreased, and even undetectable (*Cx26* in SK-HEP-1 cells), in the vast majority of the liver cancer cell lines when compared to PHH. These reductions seemed mildest in C3A and PLC/PRF/5 cells for *Cx32*, and in SNU-387, SNU-475 and PLC/PRF/5 cells for *Cx26* (Figure 2B). The exact same trends could be seen when performing RT-qPCR on cancer cell line cultures during their exponential growth phase (Figure S2A,B).

By contrast, *Cx43* mRNA abundance was significantly increased in three cell lines, namely SNU-449, SNU-387 and PLC/PRF/5 cells, compared to PHH (Figure 2C). This specifically held true for the PLC/PRF/5 cell line, which showed a 50-fold upregulation in *Cx43* production compared to PHH. *Cx43* gene expression in SNU-423 cells and SNU-475 cells was higher compared to PHH but was rather negatively affected in C3A and SK-HEP-1 cells. Again, these results were very similar to the results seen during the exponential growth phase of the liver cancer cell lines (Figure S2C).

Additionally, mRNA levels were expressed as a percentage of the corresponding glyceraldehyde-3-phosphate dehydrogenase (*GAPDH*) levels (Figure S3) to appreciate the relative levels of *Cx26*, *Cx32* and *Cx43* in the liver cancer cell lines or PHH. It was clear that *Cx26* (Figure S3A) and *Cx32* (Figure S3B) were the main connexin species in PHH and C3A, with *Cx32* levels being approximately 13 times higher than *Cx26* in PHH. For all cancer cell lines, except C3A cells, the *Cx43* levels (Figure S3C) were higher than the *Cx32* and *Cx26* expression when expressed as a percentage to *GAPDH*, indicating a clear switch in connexin expression patterns.

### 2.3. Connexin Protein Expression in Liver Cancer Cell Lines

**Figure 3:**
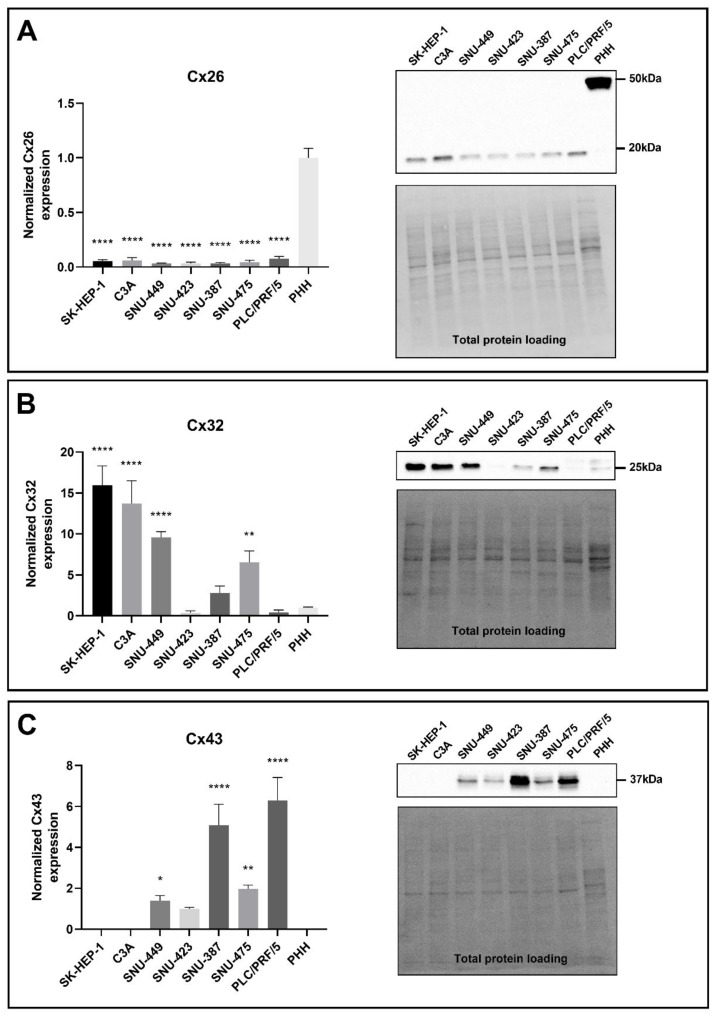
Cx26 (**A**), Cx32 (**B**) and Cx43 (**C**) protein expression in liver cancer cell lines and primary human hepatocytes (PHH). Cancer cell lines (*n* = 1, *N* = 3) were grown to 100% confluence, while PHH were used in suspension for protein extraction. Immunoblotting and visualization were done with the Pierce™ ECL Western Blotting Substrate kit (Thermo Fisher Scientific, Waltham, MA, USA) on a ChemiDoc^TM^ MP imaging system. All signals were divided by their respective total protein loading signal and normalized by the sum of all data points in a replicate [42]. Unlike Cx43, which was not expressed by PHH, Cx26 and Cx32 are expressed relative to their expression in PHH. Data are expressed as mean ± standard deviation with * *p* ≤ 0.05, ** *p* ≤ 0.01 and **** *p* ≤ 0.0001 compared to the PHH control.

A number of reports have shown differentially affected connexin protein expression in human and rat HCC tumor samples [20,21,22,38] as well as in human and rat liver cancer cell lines [38,39,40]. In this study, both Cx32 and Cx26, but not Cx43, were detected in PHH at the protein level (Figure 3A–C). Cx26 was detected at two different molecular weights, namely at 17 kDa and 50 kDa. Both molecular weights were analyzed, as the band at 50 kDa was presumed to be a dimer [41]. In accordance with the RT-qPCR data (Figure 2A), Cx26 protein moieties were decreased in all liver cancer cell lines compared to PHH (Figure 3A) when immunoblot analysis was performed on 100% confluent cell cultures. The same result was seen during the exponential growth phase of the cancer cell lines (Figure S4A).

While *Cx32* gene expression was suppressed in all liver cancer cell lines compared to PHH (Figure 2B), a downregulation at the translational level could only be observed for SNU-423 and PLC/PRF/5 cells, although not significant (Figure 3B). All other liver cancer cell lines, except for the SNU-387 cells, showed significantly increased Cx32 protein levels (Figure 3B). The same trends were seen during the exponential growth phase of the liver cancer cell lines (Figure S4B). Cx43 was detected as three different signals around 37 kDa in PLC/PRF/5 (Figure 3C). These represent the non-phosphorylated isoform, the phosphorylated isoform and the double-phosphorylated isoform from the lowest to the highest molecular weight, respectively. All bands were included in the quantification of Cx43 in all cell lines. In compliance with the mRNA profile (Figure 2C) and the protein expression during the exponential growth phase (Figure S4C), Cx43 protein production was enhanced in SNU-449, SNU-387, PLC/PRF/5, SNU-475 and SNU-423 cells compared to PHH (Figure 3C), although the latter was not significant. Cx43 was not found in C3A and SK-HEP-1 cells or PHH (Figure 3C), despite being detected during RT-qPCR analysis (Figure 2C), albeit at a very low level (not visible on graph).

### 2.4. Connexin Protein Subcellular Localization in Liver Cancer Cell Lines

**Figure 4:**
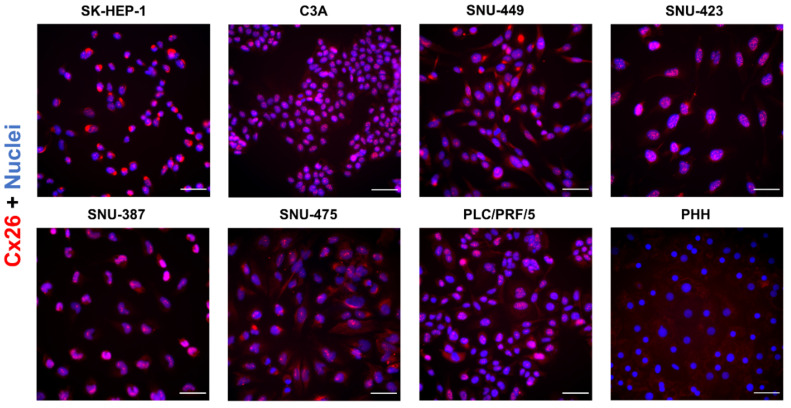
Cx26 protein localization in liver cancer cell lines and primary human hepatocytes (PHH). Cancer cell lines (*n* = 1, *N* = 2) were fixed during the exponential growth phase, while PHH were fixed at the final day of the sandwich cultivation period. All cells were immunostained for Cx26 (red) with nuclear counterstaining using 4′,6-diamidino-2-phenylindole (DAPI) (blue). Scale bar = 50 µm. Images were taken with a 40× objective. A representative image is shown.

In normal liver, approximately 3% of the hepatocellular membrane surface is occupied by Cx32-based gap junctions [43]. In general, the majority of the connexin proteins are located at the cell plasma membrane [44]. Nevertheless, a substantial amount of them are found in the cytosol, probably due to their rapid turn-over [44,45,46]. Indeed, connexin half-lives are 2–3 h [45,47], while this ranges from 17 to 100 h for most plasma membrane proteins in primary human hepatocytes [46]. In HCC, both in vitro [48,49] and in vivo [19,38,49], a shift in connexin location has been noted from the membrane to the cytoplasm. In addition, the extent of intracellular Cx43 localization has been related to the malignant potential of rat liver epithelial cell lines [39]. The immunocytochemistry data of the present study confirmed Cx26 occurrence in all liver cancer cell lines, appearing predominantly in the perinuclear areas and as a dotted signal coinciding with the signal of the nuclei (Figure 4). This was in contrast to PHH, which did not display this punctuation at the nuclei and only showed a signal in the cytoplasm.

**Figure 5:**
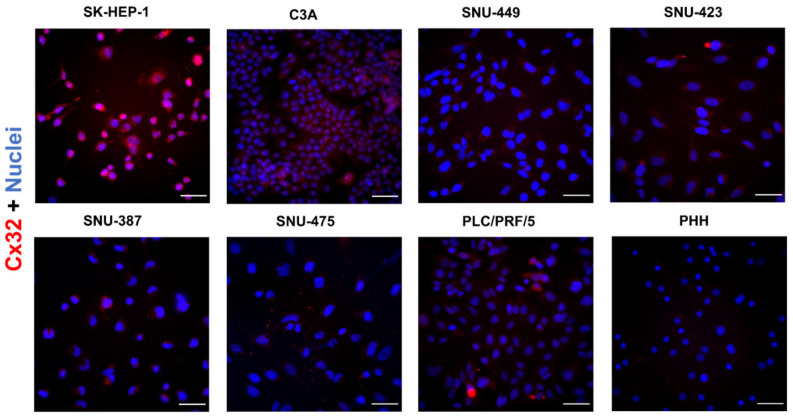
Cx32 protein localization in liver cancer cell lines and primary human hepatocytes (PHH). Cancer cell lines (*n* = 1, *N* = 2) were fixed during the exponential growth phase, while PHH were fixed at the final day of the sandwich cultivation period. All cells were immunostained for Cx32 (red) with nuclear counterstaining using 4′,6-diamidino-2-phenylindole (DAPI) (blue). Scale bar = 50 µm. Images were taken with a 40× objective. A representative image is shown.

In situ immunostaining indicated a merely low presence of Cx32 in all liver cancer cell lines and PHH (Figure 5). SK-HEP-1, C3A, SNU-423 and PLC/PRF/5 cells displayed a dotted Cx32 pattern in the nuclei and a diffuse signal in the cytoplasm.

**Figure 6:**
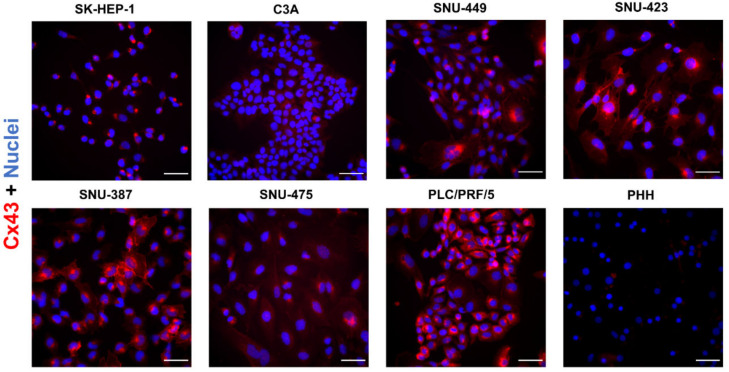
Cx43 protein localization in liver cancer cell lines and primary human hepatocytes (PHH). Cancer cell lines (*n* = 1, *N* = 2) were fixed during the exponential growth phase, while PHH were fixed at the final day of the sandwich cultivation period. All cells were immunostained for Cx43 (red) with nuclear counterstaining using 4′,6-diamidino-2-phenylindole (DAPI) (blue). Scale bar = 50 µm. Images were taken with a 40× objective. A representative image is shown.

In support of the immunoblot analysis results (Figure 3), Cx43 was most prominently represented in PLC/PRF/5 cells and the SNU cell lines but showed faint staining in C3A and SK-HEP-1 cells and PHH (Figure 6).

Unlike Cx32 and Cx26, Cx43 formed a delineated pattern in various liver cancer cell lines, which suggests predominant localization at the cell plasma membrane surface (Figure 6).

### 2.5. Gap Junctional Intercellular Communication in Liver Cancer Cell Lines

**Figure 7:**
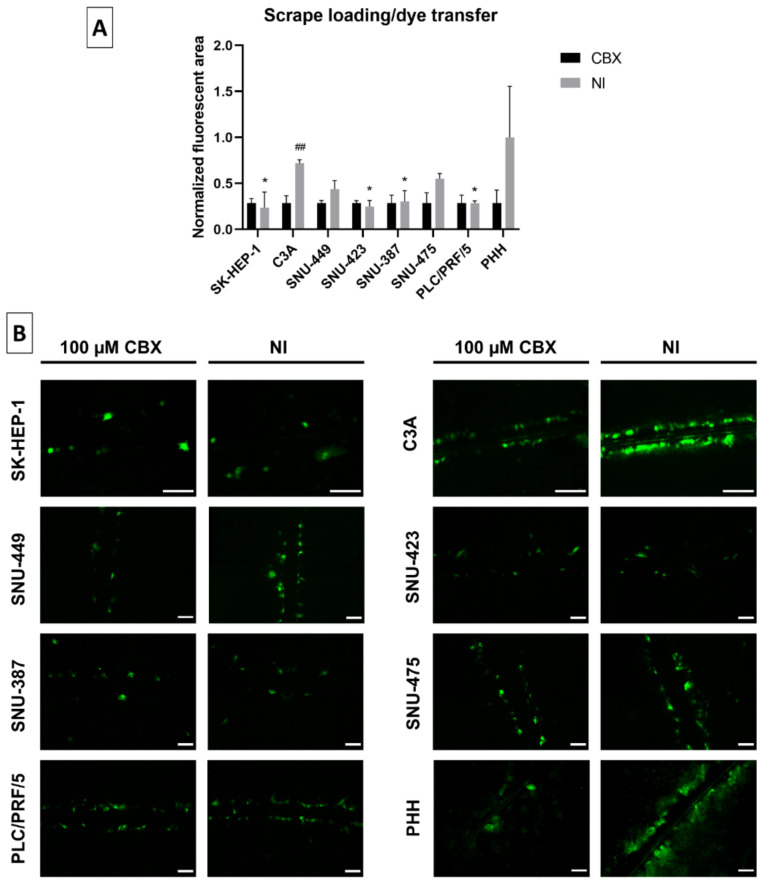
Gap junctional intercellular communication (GJIC) in liver cancer cell lines. All cell lines (*n* = 1–2, *N* = 1 (multiple images per well)) were grown to 100% confluence when a scrape was made across the well in presence of Lucifer Yellow (LY). Cells were washed and subjected to fluorescence microscopy analysis. Images were taken with a 10× or 20× objective (**B**). GJIC was either not inhibited (NI) or inhibited by 100 µM carbenoxolone disodium salt (CBX) for 10 min. The fluorescent area was measured with ImageJ. Data were normalized to the inhibited control and expressed as a ratio to the mean NI fluorescent area of primary human hepatocytes (PHH). Data are expressed as mean ± standard deviation (**A**). Significant differences compared to NI PHH are indicated with * *p* ≤ 0.05, while differences to the respective CBX control are indicated with ^##^ *p* ≤ 0.01. Scale bar = 100 µm.

A scrape loading/dye transfer assay was used to assess GJIC. A scratch was hereby made with a needle into the confluent cell layer in the presence of the fluorescent dye Lucifer Yellow (LY). The latter was taken up by the damaged cells along the scrape. When functional, LY was transferred to undamaged neighboring cells through gap junctions. The fluorescent area was used as a measure to assess GJIC. Throughout these experiments, carbenoxolone disodium salt (CBX), a well-known inhibitor of gap junctions [50], was used as a control. C3A cells displayed functional GJIC, as shown by the significant difference between the CBX-inhibited and non-inhibited (NI) cells (Figure 7A,B).

Although PHH displayed functional GJIC along certain scrapes, the higher variation in these cultures ensured that the overall GJIC was not significant compared to the inhibited control. In most cell lines, the NI fluorescent area was similar to the fluorescent area of the inhibited CBX control (Figure 7B). This indicated the absence of functional GJIC, which was also reflected by their significant lack of fluorescent dye transfer compared to NI PHH.

## 3. Discussion

Cell lines are popular experimental settings in scientific research because of a number of reasons, including ease of use and high reproducibility of testing results [5,51]. Many cell lines have a carcinogenic background and hence are frequently relied upon to test candidate anti-cancer drugs, as well as to elucidate mechanisms of carcinogenesis [51]. Among such mechanisms is cellular communication mediated by gap junctions [13,52]. Modifications in the expression of connexins in human and rodent liver cancer tissue have been reported on many occasions [21,23,24,38,53,54,55]. Several studies describe a reduction in Cx26 and Cx32 levels and a concomitant upregulation of Cx43 expression in various chronic liver diseases, most of which ultimately lead to HCC development [17]. The tumor-promoting role of Cx43 in HCC has been indicated on different occasions [56,57]. A knockdown of Cx43 production in human hepatoma cells triggers cell cycle arrest, boosts the differentiated status [56] and suppresses invasion, migration and metastasis [57], whereas inverse observations are seen in their Cx43-overexpressing counterparts [56].

To gain a broader insight into changes in connexin expression and gap junction functionality in HCC in vitro, this study subjected seven different human cell lines, commonly applied to liver cancer research, to an analysis of gene and protein expression, as well as of subcellular localization of Cx26, Cx32 and Cx43, along with testing of GJIC responses. Reminiscent of the in vivo situation [19,21,22], Cx43 de novo expression was found in various liver cancer cell lines when compared to PHH. The increase in protein abundance was hereby paralleled by almost identical changes at the mRNA level. This suggests regulation by the transcriptional machinery. In this respect, the proto-oncogenes *c*-fos and *c*-jun are known to be induced upon hepatocarcinogenesis [58]. These proteins form the transcription factor activator protein 1, which controls Cx43 expression [59]. In myometrial cells, activator protein 1 has been found to activate *Cx43* gene transcription in stress conditions [60]. This mechanism could possibly underlie Cx43 production in liver cancer cells as well. Although most cell lines supported a tumor-promoting role for Cx43, this connexin species was not detected in SK-HEP-1 and C3A cells during immunoblot analysis. Indeed, contradicting studies about the role of Cx43 in HCC have been published. Various studies proposed a downregulation or even absence of Cx43 in human HCC samples [38,53,61], in vivo rat studies [62] and human [38] and rat liver cancer cell lines [63]. Another reason for the absence of Cx43 protein expression in C3A cells could be the fact that this cancer cell line has retained a more differentiated status compared to other liver cancer cell lines and therefore does not express the Cx43 protein [27,28]. The SK-HEP-1 cell line, on the other hand, is known to originate from endothelial cells and was more recently proposed to serve as a model for liver sinusoidal cells rather than HCC, which they have been widely used for in the past [64]. While liver endothelial cells normally express Cx43 [16], SK-HEP-1 does not, according to various studies [65,66,67], which is in line with the results of the present study.

Downregulation of *Cx26* gene expression, as seen in the present study, has been equally observed in rat [68] and human HCC tissue [23] and complies with results from others using human liver cancer cell lines [38]. This has been associated with hypermethylation of the *Cx26* gene promotor [68]. Cx26 and particularly Cx32 are known to act as liver tumor suppressors [17]. In this regard, Cx32-knockout animals display elevated susceptibility for the development of liver tumors [69,70]. At the same time, overexpression of Cx32 inhibits metastasis and proliferation of liver cancer cells [71]. *Cx32* gene or protein expression has been repeatedly reported to be decreased in HCC in vivo [21,24,72,73], in vitro [38,49] and ex vivo [19,23,38,49,74]. This was also found by RT-qPCR analysis in the current study. However, with the exception of Cx32 expression in SNU-423 and PLC/PRF/5 cells, these findings were not reflected and actually contradictory at the protein level. Upregulated [53] or even unchanged [19,20,22] Cx32 protein expression has also been observed in human HCC samples. Such discrepancy between *Cx32* mRNA and protein expression has been equally seen in non-alcoholic steatohepatitis, which often leads to HCC [75] and may be associated with shortening of the poly(A) tail in *Cx32* mRNA [76,77].

GJIC deterioration is commonly observed in (chronic) liver diseases [78]. In HCC, GJIC has been inversely correlated with Cx43 expression and cell line malignancy levels [39,56]. Reduction of GJIC in HCC was supported by the results of the present study. This could represent an escape from homeostasis, this being a hallmark of cancer [13]. The lack of GJIC in SK-HEP-1 cells has been previously reported [79]. Nevertheless, gap junction activity could be detected in some liver cancer cell lines, in particular C3A cells and to a lesser extent in SNU-449 and SNU-475 cells.

In summary, the results of this study provide for the first time a full characterization of in vitro modifications in connexin expression as well as gap junction activity in liver cancer cell lines. PLC/PRF/5 is the cell line that stands out the most for displaying a decrease in Cx26 and Cx32 on both the transcriptional and translational levels, together with an increase in Cx43 on both levels and a reduction of GJIC. Although the presented data are based on single extractions and should be confirmed in follow-up studies, these findings may be of great relevance for the selection of liver cancer cell lines for future mechanistic investigation and testing of anti-cancer drugs that target connexins and their channels.

## 4. Materials and Methods

### 4.1. Reagents

Dimethyl sulfoxide (DMSO), CBX and LY were supplied by Sigma-Aldrich (St. Louis, MO, USA). All other reagents were obtained from various suppliers at the highest analytical grade possible.

### 4.2. Liver Cancer Cell Lines and Primary Human Hepatocytes

PHH were purchased from KaLy-cell (Plobsheim, France) and cultured in a sandwich culture for scrape loading/dye transfer analysis and immunocytochemistry analysis. PHH were thawed and transferred to a tube with prewarmed cryopreserved hepatocyte recovery medium (CHRM) (70001, APSciences, Columbia, MD, USA). After gentle mixing, PHH were pelleted by centrifugation at 100× *g* for 10 min at room temperature. Cell culture medium was discarded, and the PHH pellet was resuspended in 2–5 mL cold cryopreserved hepatocyte plating medium (CHPM) (70002, APSciences, Columbia, MD, USA) depending on the pellet size. A total of 0.36 × 10^6^ cells were seeded per well in a 24-well plate coated with rigid rat tail collagen I (Corning, NY, USA). An amount of 0.1 mg/mL collagen was dissolved in 0.02 N acetic acid (Sigma-Aldrich, St. Louis, MO, USA) for coating of the plates before cell seeding. Plates were incubated at 37 °C with 5% CO_2_ and 95% humidity. At least 4 h after seeding, the cell culture medium was replaced with warm cryopreserved hepatocyte plating medium. Twenty-four hours after seeding, the monolayer was washed with Dulbecco’s phosphate-buffered saline (PBS) (Gibco, Waltham, MA, USA), and a Matrigel^®^ (Corning, NY, USA) overlay was added. Matrigel^®^ was dissolved in maintenance medium [Williams E medium (A12176-01, Gibco, Waltham, MA, USA), 1% L-glutamine–penicillin–streptomycin (Sigma-Aldrich, St. Louis, MO, USA), 1% insulin–transferrin–selenium (Gibco, Waltham, MA, USA) and 0.01% dexamethasone (Sigma-Aldrich, St. Louis, MO, USA) dissolved at 1 mM in DMSO] at a concentration of 0.25 mg/mL. Maintenance medium was replaced 2 h later, after gelatinization of the Matrigel^®^ layer at 37 °C, and again 2 days after addition of the Matrigel^®^ layer. PHH sandwich cultures were maintained for 4 days after seeding.

The Liver Cancer Panel (ATCC^®^ TCP-1011™) was purchased from the American Type Culture Collection (ATCC, Manassas, VA, USA) and contained 7 human liver cancer cell lines, namely SK-HEP-1, C3A, SNU-449, SNU-423, SNU-387, SNU-475 and PLC/PRF/5 (Table 1). All cell lines were cultured at 37 °C with 5% CO_2_ and 95% humidity. Cells were grown according to the supplier’s recommendations and were used at 100% confluence unless specified otherwise. Cells were grown in Roswell Park Memorial Institute 1640 Medium (ATCC 30-2001™, Manassas, VA, USA or A1049101, Gibco, Waltham, MA, USA) or Eagle’s Minimum Essential Medium (EMEM)(ATCC 30-2003™, Manassas, VA, USA or Minimum Essential Medium (MEM) (11095080, Gibco, Waltham, MA, USA) with addition of 1% MEM Non-Essential Amino Acids Solution (11140035, Gibco, Waltham, MA, USA) and 1% Sodium Pyruvate (11360039, Gibco, Waltham, MA, USA) supplemented with 10% fetal bovine serum (FBS) (ATCC, USA) (heat-inactivated for SNU-475, SNU-449 and SNU-423) and 100 units/mL penicillin and 100 µg/mL streptomycin (Thermo Fisher Scientific, Waltham, MA, USA).

### 4.3. Phase-Contrast Microscopy

Microscopy pictures were taken during the exponential growth phase of the cell lines and at 100% confluence with a Nikon Eclipse Ti-S microscope (Nikon, Tokyo, Japan) using a 10× objective. PHH sandwich cultures were imaged daily on the same microscope with a 20× objective. Scale bars were added with ImageJ software (version 1.52p) (Bethesda, MD, USA).

### 4.4. Real-Time Quantitative Reverse Transcription Polymerase Chain Reaction Analysis

mRNA expression analysis was carried out as previously described with minor modifications [44]. In essence, total RNA was extracted from cultured cell lines and freshly thawed PHH using a GenElute^TM^ Mammalian Total RNA Miniprep Kit (Sigma-Aldrich, St. Louis, MO, USA) combined with an On-Column DNase I Digestion Set (Sigma-Aldrich, St. Louis, MO, USA) according to the manufacturer’s instructions. RNA yield and purity were assessed using a NanoDrop 2000 spectrophotometer (Thermo Fisher Scientific, Waltham, MA, USA). Next, cDNA was generated with an iScript™ cDNA Synthesis Kit (Bio-Rad, Hercules, CA, USA) on a MiniAmp Plus Thermal Cycler (Thermo Fisher Scientific, Waltham, MA, USA) followed by cDNA purification using a GenElute™ PCR Clean-Up Kit (Sigma-Aldrich, St. Louis, MO, USA). RT-qPCR analysis was performed on an Applied Biosystems QuantStudio 3 Real-Time PCR system (Thermo Fisher Scientific, Waltham, MA, USA). Samples were tested in triplicate and amplified with TaqMan^®^ Gene Expression Assays (Applied Biosystems, Waltham, MA, USA) targeted towards 5 housekeeping genes, namely actin beta (*ACTB*), beta-2-microglobulin (*B2M*), *GAPDH*, hydroxymethylbilane synthase (*HMBS*) and ubiquitin C (*UBC*), as well as towards *Cx26*, *Cx32* and *Cx43* (Table 2).

The reaction mix contained 2 µL cDNA, 1 µL primer, 7 µL nuclease-free water and 10 µL of TaqMan^®^ Fast Advanced Master Mix (Applied Biosystems, Waltham, MA, USA). Efficiency was estimated based on a serial dilution of pooled cDNA mix from all samples and 3 no-template controls. Relative alterations compared to PHH were calculated according to the Pfaffl method [80] in qbase^+^ (Biogazelle, Gent, Belgium).

### 4.5. Immunoblot Analysis

Immunoblot analysis was performed as described previously with small modifications [81]. Cell lines were washed twice with ice-cold PBS and harvested by scraping in the presence of radio-immunoprecipitation assay buffer (Thermo Fisher Scientific, Waltham, MA, USA) supplemented with 1% ethylenediaminetetraacetic acid (Thermo Fisher Scientific, Waltham, MA, USA) and 1% protease and phosphatase inhibitor cocktail (Thermo Fisher Scientific, Waltham, MA, USA). PHH were not cultured for protein extraction but were freshly thawed and washed twice with ice-cold PBS. After centrifugation for 5 min at 4 °C at 2060× *g*, the resulting pellet was resuspended in the same protein lysis buffer as the cell lines. Next, lysates were transferred to a pre-cooled tube and put on ice for 30 min, during which the lysate was vortexed every 5 min. Supernatants were collected by centrifugation for 20 min at 4 °C and 14,000× *g* and stored at −80 °C. Protein concentrations were determined with the Pierce^TM^ BCA protein assay kit (Thermo Fisher Scientific, Waltham, MA, USA). For immunoblotting, proteins were separated on 10% (Cx43) or 12% (Cx26 and Cx32) Mini-PROTEAN TGX Stain-Free™ precast gels (Bio-Rad, Hercules, CA, USA). Samples for detection of Cx26 were heated for 5 min at 95 °C. Gels were blotted onto a nitrocellulose membrane (Cx43 and Cx32) (Bio-Rad, Hercules, CA, USA) or a polyvinylidene difluoride membrane (Cx26) (Bio-Rad, Hercules, CA, USA). Blocking of the membranes was performed in 5% non-fatty milk in Tris-buffered saline solution (20 mM Tris and 135 mM sodium chloride) with 0.1% Tween-20 for 1 h at room temperature on an orbital shaker. Membranes were incubated overnight at 4 °C with primary antibodies raised against Cx26, Cx32 or Cx43 (Table 3) diluted in blocking buffer.

Membranes were washed 3 times for 10 min and incubated with a secondary antibody (Cx26 and Cx32: 1:500; Cx43: 1:2000, P0448, Dako, Næstved, Denmark) diluted in blocking buffer for 1 h at room temperature. Membranes were washed and visualized by using the Pierce™ ECL Western Blotting Substrate kit (Thermo Fisher Scientific, Waltham, MA, USA) on a ChemiDoc^TM^ MP imaging system (Bio-Rad, Hercules, CA, USA). Densiometric analysis was performed with Image Lab 6.0.1 software (Bio-Rad, Hercules, CA, USA). Signals were normalized against total protein loading and by sum of all data points in a replicate [42]. Data are expressed as fold change relative to the corresponding signals in PHH, unless mentioned otherwise.

### 4.6. Immunocytochemistry Analysis

Cell lines and PHH cultures were washed 3 times with ice-cold PBS for 5 min and fixed at −20 °C for 10 min with an acetone–ethanol (1:1) mixture that permeabilized simultaneously. Cells were then washed 3 times for 5 min with ice-cold PBS. Cells were blocked at room temperature with 1% bovine serum albumin (BSA)/5% donkey serum for 45 min and incubated overnight at 4 °C with primary antibodies. Primary antibodies targeted against Cx26, Cx32 and Cx43 (Table 3) were diluted in 1% BSA dissolved in PBS. Excess of antibody was washed away 3 times with PBS for 5 min. This was followed by incubation with a secondary antibody (1:500, Alexa Fluor 594-AffiniPure Donkey Anti-Rabbit IgG, 711-585-152, Jackson ImmunoResearch, West Grove, PA, USA) diluted in 1% BSA dissolved in PBS for 1 h at room temperature. Cells were again washed 3 times for 5 min with PBS and 1 time with double-distilled water. Nuclei were stained by adding VECTASHIELD Antifade Mounting Medium with 4′,6-diamidino-2-phenylindole (DAPI) (Vector Laboratories, Burlingame, CA, USA). Detection was performed on a Nikon Eclipse Ti microscope (Nikon, Tokyo, Japan) with a 40× objective. Image editing was done with ImageJ software (version 1.52p) (Bethesda, MD, USA).

### 4.7. Scrape Loading/Dye Transfer Assay

Cell lines and PHH were washed 3 times with scrape loading/dye transfer (SL/DT) buffer (137 mM NaCl, 5.36 mM KCl, 0.8 mM MgCl_2_, 5.55 mM glucose and 25 mM 4-(2-hydroxyethyl)-1-piperazineethanesulfonic acid (HEPES); pH 7.4) or CBX buffer (100 µM CBX in SL/DT buffer). Next, a scratch was made with a fine needle in the presence of LY (1 mg/mL) dissolved in SL/DT or CBX buffer. This was incubated for 10 min at room temperature. Then, cells were washed 4 times with Hank’s Balanced Salt Solution (HBSS)/ HEPES buffer (0.95 mM CaCl_2_.2H_2_0, 0.81 mM MgSO_4_.7H_2_O, 137 mM NaCl, 5.36 mM KCl, 5.55 mM glucose and 25 mM HEPES; pH 7.4) and fixed for 10 min in 4% paraformaldehyde solution. Cells were washed again 4 times with HBSS/HEPES buffer and visualized on a Nikon Eclipse Ti-S microscope (Nikon, Tokyo, Japan) with a 10× or 20× objective. The fluorescent area of the dye spread was measured via ImageJ software (version 1.52p) (Bethesda, MD, USA). Data were normalized to the area of the inhibited cells and expressed as a ratio to the non-inhibited signals in PHH.

### 4.8. Statistical Analysis

One batch was used for PHH and all cell lines (*n* = 1). The number of technical replicates (*N*) is specified in the figure legends. Statistical analysis was performed in GraphPad Prism v9.0 software (GraphPad Software, San Diego, CA, USA), and data are presented as mean ± standard deviation. Normality was assessed by means of a Shapiro–Wilk normality test. Normally distributed RT-qPCR and immunoblot data were analyzed via an ordinary 1-way analysis of variance, followed by a Dunnett’s multiple comparisons test. Scrape loading/dye transfer data were analyzed in the same way when comparing the dye transfer of the cell lines to the dye transfer of the PHH. For comparing the inhibited to the non-inhibited fluorescent area within each cell line, multiple unpaired *t*-tests were performed with a Holm–Šidák correction for multiple comparisons. Significance levels are indicated by to the following symbols: * *p* ≤ 0.05, ** *p* ≤ 0.01, *** *p* ≤ 0.001 and **** *p* ≤ 0.0001.

## Figures and Tables

**Table 1:** Overview of the liver cancer cell lines and primary human hepatocytes (PHH). Specifications for all cell types used in this study, including morphology, disease, age, gender, ethnicity, hepatitis B virus (HBV) infection of the donors and used cell culture medium (HCC, hepatocellular carcinoma; EMEM, Eagle’s minimum essential medium; FBS, fetal bovine serum; RPMI-1640, Roswell Park Memorial Institute 1640 Medium; CHRM, cryopreserved hepatocyte recovery medium; CHPM, cryopreserved hepatocyte plating medium).

Cell Line	Morphology	Disease	Age	Gender	Ethnicity	HBV	Medium
SK-HEP-1	Epithelial	Adenocarcinoma	52	Male	Caucasian	Not detected	EMEM + FBS
C3A	Epithelial	HCC	15	Male	Caucasian	Not detected	EMEM + FBS
SNU-449	Epithelial	Grade II-III/IV, HCC	52	Male	Asian	DNA detected	RPMI-1640 + heat-inactivated FBS
SNU-423	Epithelial	Grade III/IV, pleomorphic HCC	40	Male	Asian	DNA detected	RPMI-1640 + heat-inactivated FBS
SNU-387	Epithelial	Grade IV/V, pleomorphic HCC	41	Female	Asian	DNA detected	RPMI-1640 + FBS
SNU-475	Epithelial	Grade II-IV/V, HCC	43	Male	Asian	DNA detected	RPMI-1640 + heat-inactivated FBS
PLC/PRF/5	Epithelial	HCC	24	Male	African	Secreted HbsAg	EMEM + FBS
PHH	Epithelial	Liver metastasis of colorectal adenocarcinoma	74	Female	Caucasian	Not detected	CHRM + CHPM

**Table 2:** Primers and probes for real-time quantitative reverse transcription polymerase chain reaction (RT-qPCR) analysis. Assay identification (ID), accession number, assay location, amplicon size and exon boundaries are listed (*GJA1*, Cx43; *GJB1*, Cx32; *GJB2*, Cx26; *ACTB*, actin beta; *B2M*, beta-2-microglobulin; *GAPDH*, glyceraldehyde-3-phosphate dehydrogenase; *HMBS*, hydroxymethylbilane synthase; *UBC*, ubiquitin C).

Gene Symbol	Assay ID	Accession Number	Assay Location	Amplicon Size (Base Pairs)	Exon Boundary
*GJA1 (* *Cx43* *)*	*Hs*00748445-s1	NM_000165.4	1031	142	2
*GJB1 (* *Cx32* *)*	*Hs*00939759-s1	NM_000166.5 NM_001097642.2	1547 1496	63	2
*GJB2 (* *Cx26* *)*	*Hs*00269615-s1	NM_004004.5	715	123	2
*ACTB*	*Hs*01060665-g1	NM_001101.3	208	63	2–3
*B2M*	*Hs*00187842-m1	NM_004048.2	134	64	1–2
*GAPDH*	*Hs*02786624-g1	NM_001256799.2 NM_001289745.1 NM_001289746.1 NM_002046.5	870 928 822 836	157	7 8 7 8
*HMBS*	*Hs*00609296-g1	NM_000190.3 NM_001024382.1 NM_001258208.1 NM_001258209.1	1070 972 950 1041	69	13–14 13–14 12–13 12–13
*UBC*	*Hs*01871556-s1	M26880.1	2173	135	–

**Table 3:** Overview of primary antibodies. Specifications regarding the primary antibodies used in this study, including target, product code, dilution used in immunoblot analysis and immunostaining analysis and the supplier.

Target	Product Code	Dilution Primary Antibody		Supplier
		Immunoblot	Immunocytochemistry	
Cx26	51–2800	1:250	1:250	Invitrogen, Waltham, MA, USA
Cx32	C3470	1:600	1:500	Sigma-Aldrich, St. Louis, MO, USA
Cx43	C6219	1:4000	1:1000	Sigma-Aldrich, St. Louis, MO, USA

## Data Availability

Data are available upon request.

## Supplementary Materials

The following are available online at https://www.mdpi.com/article/10.3390/ijms222212187/s1.
