# Supplementary material for: Expression and Functionality of Connexin-Based Channels in Human Liver Cancer Cell Lines
Source: Int J Mol Sci. 2021 Nov 10;22(22):12187. doi: 10.3390/ijms222212187 (PMC8623148; doi:10.3390/ijms222212187)
Supplement: Supplementary file 1 [file ijms-22-12187-s001.zip › ijms-1428828-supplementary.pdf]

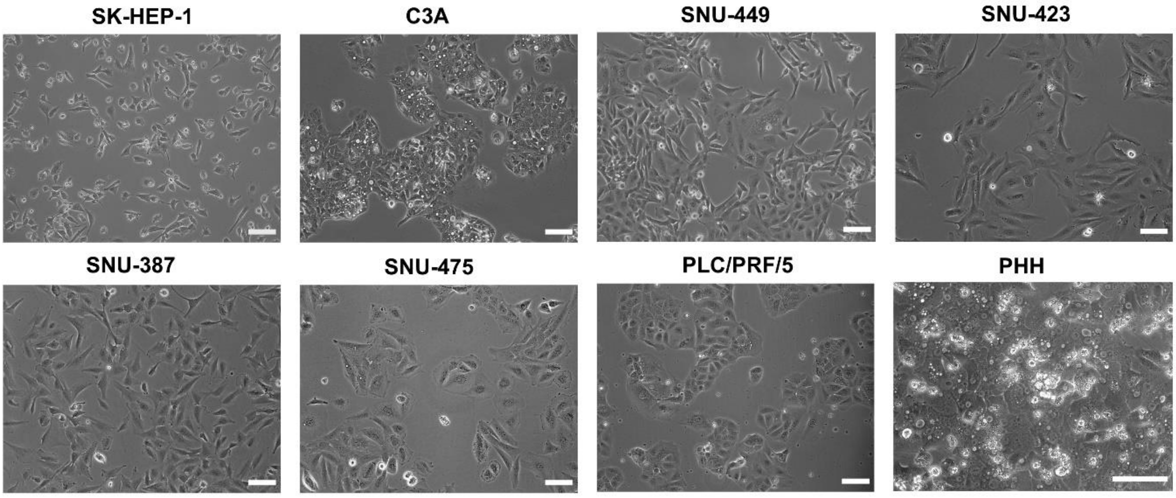


**Figure S1.** Morphology of liver cancer cell lines and primary human hepatocytes (PHH). Morphology images of all cancer cell lines were taken during the exponential growth phase. PHH sandwich cultures were imaged at the last culture day. Images were taken with a 10× or 20× objective on a Nikon Eclipse T*i*-S microscope (Nikon, Tokyo, Japan). Scale bar = 100μm.

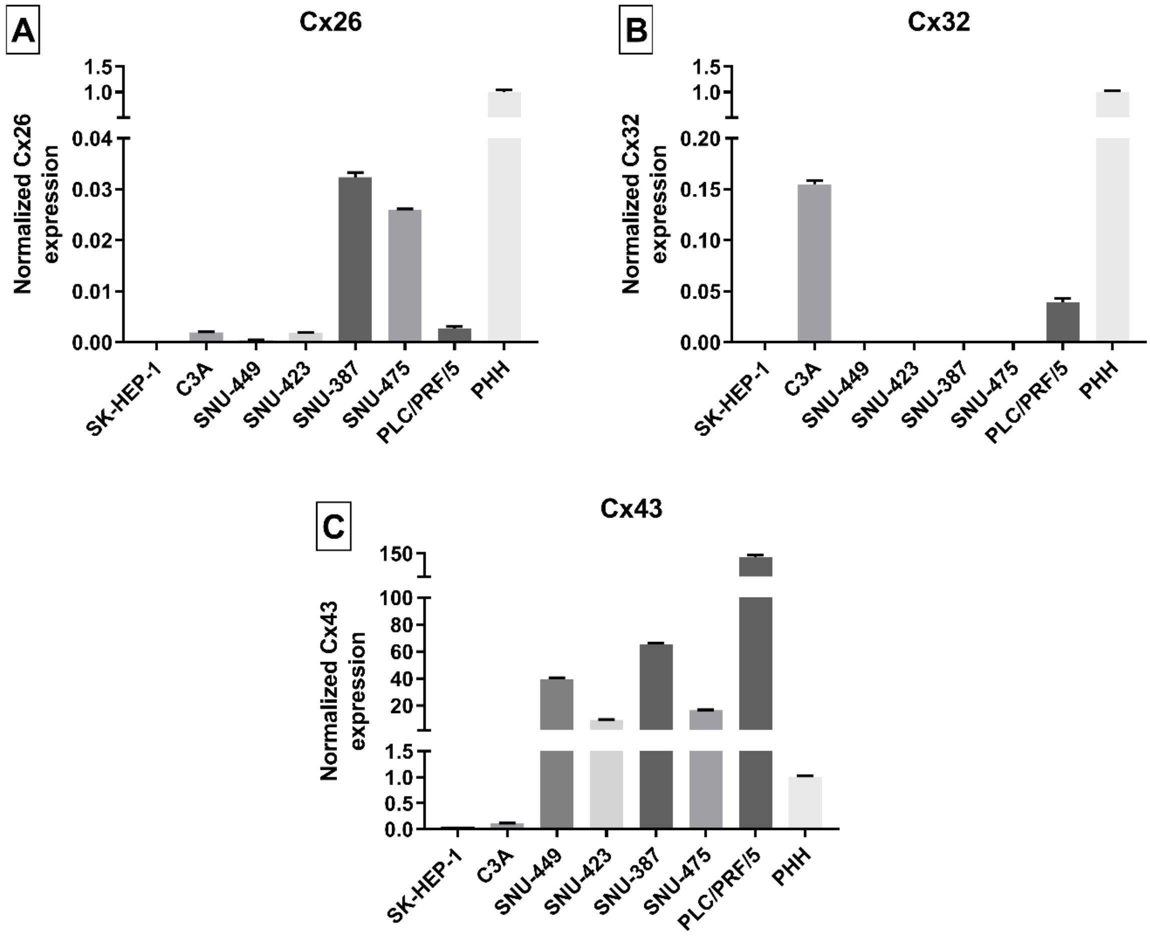


**Figure S2.** Preliminary data of *Cx26* (**A**), *Cx32* (**B**) and *Cx43* (**C**) gene expression in liver cancer cell lines and primary human hepatocytes (PHH) during the exponential growth phase of the liver cancer cell lines. Cancer cell lines ($n$ = 1, $N$ = 2) were grown to 60–90% confluence, while PHH ($n$ = 1, $N$ = 2) were used in suspension when total RNA was extracted. Subsequently, real-time quantitative reverse transcription polymerase chain reaction (RT-qPCR) analysis was performed. Relative alterations compared to PHH were calculated according to the Pfaffl formula in qbase+ (Biogazelle, Gent, Belgium).

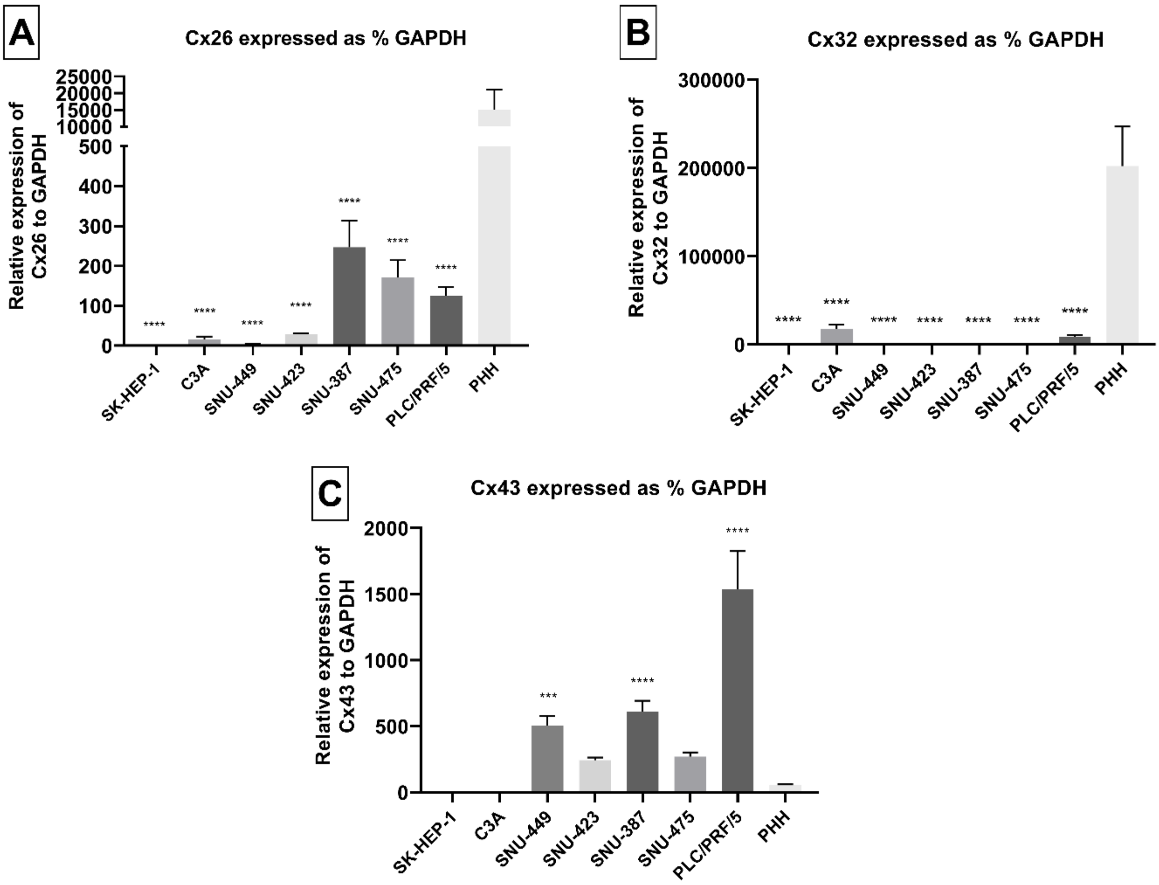


**Figure S3.** *Cx26* (**A**), *Cx32* (**B**) and *Cx43* (**C**) gene expression in liver cancer cell lines and primary human hepatocytes (PHH) expressed as ratio to glyceraldehyde-3-phosphate dehydrogenase (*GAPDH*). Cancer cell lines ($n = 1$, $N = 3$) were grown to 100% confluence, while PHH were used in suspension when total RNA was extracted. Subsequently, real-time quantitative reverse transcription polymerase chain reaction (RT-qPCR) analysis was performed. Relative alterations compared to their respective *GAPDH* expression were calculated according to the Pfaffl formula in qbase+ (Biogazelle, Gent, Belgium).

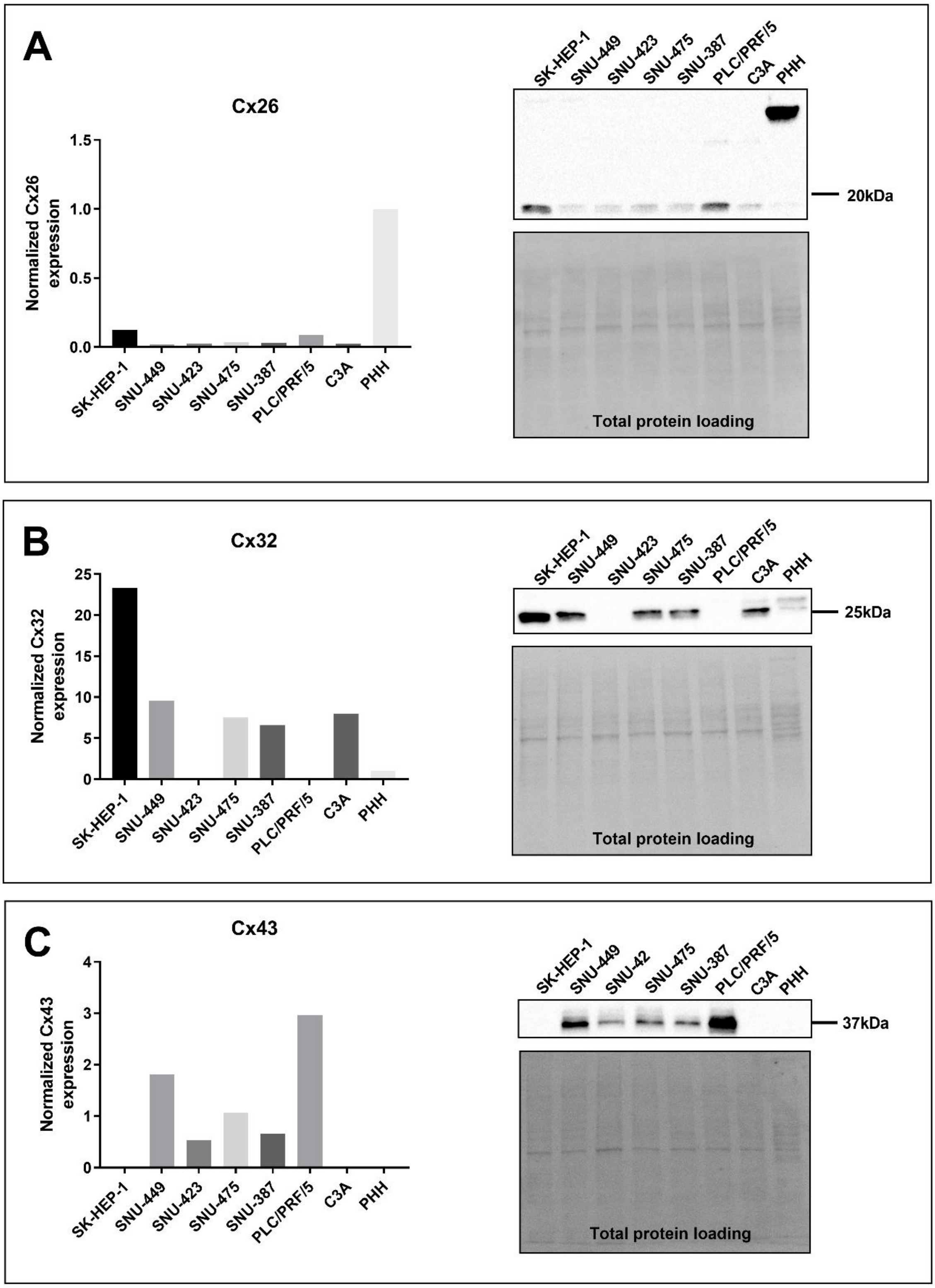


**Figure S4.** Preliminary data of Cx26 (**A**), Cx32 (**B**) and Cx43 (**C**) protein expression in liver cancer cell lines and primary human hepatocytes (PHH) during the exponential growth phase of the liver cancer cell lines. Cancer cell lines ($n = 1$, $N = 1$) were grown to 60–90% confluence while PHH were used in suspension. Proteins were extracted and quantified. Immunoblotting and visualization were done with the Pierce™ ECL Western Blotting Substrate kit (Thermo Fisher Scientific, Waltham, MA, USA) on a ChemiDocTM MP imaging system (Bio-Rad, Hercules, CA, USA). All signals were normalized to total protein loading. Unlike Cx43, which was not expressed by PHH, Cx26 and Cx32 were expressed relative to their expression in PHH.
